# Adrenal Insufficiency in Coronavirus Disease 2019 (COVID-19)-Infected Patients without Preexisting Adrenal Diseases: A Systematic Literature Review

**DOI:** 10.1155/2021/2271514

**Published:** 2021-09-14

**Authors:** Mehrbod Vakhshoori, Maryam Heidarpour, Niloofar Bondariyan, Niyousha Sadeghpour, Zohreh Mousavi

**Affiliations:** ^1^Heart Failure Research Center, Isfahan Cardiovascular Research Institute, Isfahan University of Medical Sciences, Isfahan, Iran; ^2^Isfahan Endocrine and Metabolism Research Center, Isfahan University of Medical Sciences, Isfahan, Iran; ^3^Department of Clinical Pharmacy, School of Pharmacy, Shiraz University of Medical Sciences, Shiraz, Iran; ^4^Metabolic Syndrome Research Center, Mashhad University of Medical Sciences, Mashhad, Iran

## Abstract

**Background:**

Coronavirus disease 2019 (COVID-19) manifestations varied completely from its time of emergence. However, the assessment of adrenal insufficiency (AI) in this pandemic is lacking. In this review, we aimed to evaluate the status of AI among COVID-19-infected individuals.

**Methods:**

A systematic literature screening in PubMed/MEDLINE, Scopus, and Web of Science was performed until May 23, 2021. We collected relevant published peer-reviewed studies that reported AI occurrence in patients who suffered from COVID-19.

**Results:**

A total of 10 records (cross-sectional studies: 3, *N* = 256, males: 176 (68.7%), and case reports: 7, *N* = 7, males: 4 (57.1%)) were recruited. The age spectrum ranged from 22 to 96 years. AI was diagnosed with laboratory assessment or radiologic findings. The AI prevalence ranged from 3.1% to as high as 64.3% in different studies. Except for one patient, all other patients were discharged in stable conditions in published case reports.

**Conclusion:**

This review indicates that AI occurrence in the COVID-19 pandemic seems quite probable; however, the extent and type (primary, secondary, and functional) need to be clarified yet. Appropriate early diagnostic and therapeutic interventions should be done, especially in critically ill patients, to prevent lethal outcomes.

## 1. Introduction

Coronaviridae family is a positively charged enveloped single-stranded RNA virus [1]. This family consists of three main members, including severe acute respiratory syndrome (SARS), Middle East respiratory syndrome (MERS), and Coronavirus Disease 2019 (COVID-19) [2–4]. The latter was first diagnosed in Wuhan, China, and due to its rapid spread all over the globe, the World Health Organization (WHO) declared COVID-19 as a worldwide pandemic on March 13, 2020 [5].

Till now, as in May 24, 2021, more than 166 million persons are infected with this highly contagious virus, and more than 3 million patients were deceased in this regard [6]. From the beginning of the emergence of COVID-19, most patients presented with pulmonary complaints including cough, fever, dyspnea, and respiratory distress, but this virus is not solely invading one particular tissue, and all other vital organs, including the heart, central nervous system, liver, and kidneys could also get involved [7–12]. In addition to mentioned common organs, this infection could be presented with rare manifestations, including rhabdomyolysis, hypocalcemia, and diabetic ketoacidosis [13, 14]. One probable explanation for this widespread involvement might be due to angiotensin-converting enzyme 2 (ACE2) receptors presented in other organs, in addition to the respiratory tissues [15]. The endocrine system, especially adrenal glands, might also be vulnerable during COVID-19 despite less investigation [16]. The involvement of these organs might lead to a catastrophic outcome. Till now, some case reports have been published in patients who experienced adrenal insufficiency (AI) in the context of COVID-19, but a thorough investigation in literature seems required [17, 18].

In this review, we aimed to evaluate the proportion of AI among COVID-19-infected patients.

## 2. Materials and Methods

### 2.1. Study Protocol and Registration

This review was conducted in the context of Preferred Reporting Items for Systematic Reviews and Meta-Analysis (PRISMA) [19]. We also registered the current research in the International Prospective Register of Systematic Reviews (PROSPERO) with registration ID of CRD42021255298.

### 2.2. Search Strategy

The three most common medical databases, including PubMed/MEDLINE, Scopus, and Web of Science, were screened to gather relevant records. The following search strategy was used with no time and language limitations in all aforementioned electronic databases: (“adrenal insufficiency” OR “adrenal failure” OR “adrenal injury” OR “adrenal damage” OR “adrenal^*∗*^” OR “Addison's disease” OR “Addison^*∗*^”) AND (“coronavirus” OR “covid-19” OR “SARS-CoV-2” OR “2019-nCoV” OR “novel coronavirus” OR “nCoV” OR “coronavirus covid-19”).

### 2.3. Inclusion and Exclusion Criteria

All published peer-reviewed studies (case reports, case series, cross-sectional, cohort, case-control, and review studies) investigating AI in patients with documented COVID-19 infection were assessed. Animal studies or any records with incomplete desired information or non-peer-reviewed articles were excluded.

### 2.4. Selection Process

**Figure 1:**
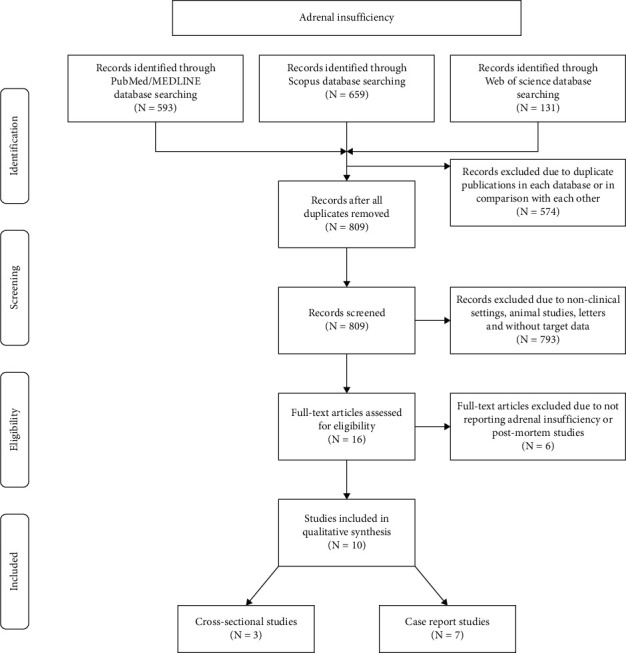
Flow diagram of current review.

Two reviewers screened the three previously mentioned electronic databases with a predefined search strategy. In terms of any relevant title or abstract, the full texts were obtained for further assessment. We only counted a single article in terms of duplication.  Figure 1 shows the flow diagram of the current review.

### 2.5. Data Extraction

Data including the first author's name, publication date, study location, and design, sample size, age (mean ± standard deviation or median (interquartile range or ranges), as reported), sex (male/female), presenting symptoms, AI assessment (laboratory data or radiographic findings), AI types (primary/central), and frequency of AI (%) were extracted based on study designs. Another researcher evaluated the whole process, and the consensus was made among all authors in terms of any discrepancies.

### 2.6. Quality and Risk of Bias Assessment

Assessment of multiple systematic reviews (AMSTAR) and critical appraisal tool (AXIS tool) were used for evaluating the quality of systematic reviews and cross-sectional studies, respectively [20, 21]. In order to assess case reports/series, the Joanna Briggs Institute (JBI) critical appraisal checklist was utilized [22]. We also evaluated the quality of case-control studies using the National Institutes of Health (NIH) quality assessment tool [23].

### 2.7. Data Synthesis and Statistical Analysis

Due to the small number of heterogeneous observational studies and some published case reports, we could not conduct a meta-analysis, and a narrative data synthesis was performed.

## 3. Results

We found 809 records in all predefined databases. After removing duplicates and implementing inclusion and exclusion criteria, 10 articles (cross-sectional studies: 3, case reports: 7) were enrolled for the current review (Figure 1). AI was defined based on laboratory data, computed tomography (CT) results, or a combination of both methods [17, 18, 24, 25]. A summary of included studies based on their designs is provided in Tables 1 and 2. Quality and risk of bias assessment of recruited records are shown in the supplementary material (Tables S1 and S2).

### 3.1. Cross-Sectional Studies

Three studies on 256 patients were performed to assess the proportion of AI among COVID-19-infected patients. Alzahrani et al. recruited 28 consecutive patients with a median age of 45.5 (range: 25, 69) years and evaluated morning blood cortisol and adrenocorticotropic hormone (ACTH). The median cortisol and ACTH means were 196 (range: 31, 587) nmol/l and 18.5 (range: 4, 38) ng/l, respectively. They also measured cortisol for the second time (3–5 days after hospitalization) on 20 patients. They found that 2 (10%), 6 (30%), and 9 (45%) enrolled participants had cortisol levels of less than 100, 200, and 300 nmol/l, respectively. ACTH was not measured for the 2^nd^ time. Fifteen patients were tested for the third time 8–11 days after admission with median cortisol and ACTH of 238 (range: 57, 594) nmol/l and 16.5 (3.1, 50.2) ng/dl, respectively. Nine (60%) participants showed cortisol means of <300 nmol/l. Furthermore, an ACTH level of less than 30 ng/dl was observed among 10 (66.6%) subjects. They finally reported that adrenocortical response during COVID-19 infection might be interrupted, and patients might suffer from central AI [25]. Leyendecker and colleagues recruited 219 patients with moderate to severe COVID-19 infection from March 9 to April 10, 2020, and retrospectively assessed their adrenal glands through triage CT scans to evaluate the incidence of acute adrenal infarction. Enlargement of at least one gland plus fat stranding in suprarenal location was defined as acute adrenal infarction. 51 (23%) subjects (males: 36 (71%), age: 67 ± 11 years) were revealed as having acute adrenal infarction based on their definitions. AI in this group was diagnosed based on biological data in 4 (7.8%) subjects. The frequency of AI in patients with no presentation of acute adrenal infarction on CT findings was 3 (1.7%), with an overall prevalence of 3.1% (7 out of 219 cases). They also suggested that these adrenal radiographic findings might be associated with a higher intensive care unit admission rate and longer hospital stay in COVID-19 patients [26]. Mao et al. recruited 9 COVID-19-infected patients and 12 non-COVID-19 patients in critical status and assessed the differences in plasma cortisol levels between these two groups. They found that cortisol was significantly lower among COVID-19 patients with severe disease (*P* < 0.01). Half of the non-COVID-19 subjects had elevated cortisol levels compared to 66.6% of patients in the COVID-19 group who showed cortisol means of <10 *µ*g/dl, which had the required criteria for diagnosis of functional AI, named critical illness-related corticosteroid insufficiency (CIRCI) [27].

### 3.2. Case Report Studies

Seven records reported AI occurrence during COVID-19 infection in 7 patients. The ages ranged from 44 to 70 years (males: 4). Evaluation of AI was done through CT findings, laboratory profiles, or both in 2 [24, 28], 3 [17, 18, 31], and 2 [29, 30] patients, respectively. Only one patient died [28], and all 6 others were discharged in stable condition with appropriate medical treatment [17, 18, 24, 29–31]. 2 patients were followed up after hospital discharge with normal status [24, 29]. However, two other subjects failed to attend follow-up visits [17, 31]. The follow-up data of two patients were not available [18, 30].

## 4. Discussion

This review was conducted to assess reported COVID-19-induced AI in the literature. In addition to several case reports, we found that some observational studies also reported this presentation, and either primary or central AI in the context of COVID-19 is not just a rare manifestation of this pandemic. It seems that the occurrence of this entity is probable in clinical settings, and the implementation of future large-scale studies to assess the exact incidence of AI is mandatory. Since the official declaration of this highly infectious agent, variable presentations in different organs have been reported [7, 8, 10–12]. One of the main mechanisms of multiple tissue involvement might be related to cell entry receptors. ACE2 receptors have been suggested to be expressed by the hypothalamus, pituitary, and adrenal glands. Because of the presence of this receptor as one of the main entry cites, COVID-19 could directly invade mentioned organs resulted in primary or secondary AI incidence [32]. Direct invasion of COVID-19 to adrenal glands has been assessed in postmortem studies. Four autopsy studies evaluated adrenal pathologic findings among deceased subjects from COVID-19. Autopsy analysis in 3 out of 9 dead individuals showed adrenal microinfarction [33]. Iuga et al. assessed adrenal glands pathological slides of 5 postmortem subjects (males: 4) aged 59 to 90. Microscopic findings favored acute fibrinoid necrosis of arterioles in adipose tissue adjacent to adrenals and parenchyma as well as the capsule of adrenal glands [34]. Another autopsy finding performed by Santana and colleagues on 28 deceased adrenal glands showed 12 (46%) cases had some extents of ischemic and hemorrhagic necrosis, cortical lipid degeneration, and focal adrenalitis [35]. Zinserling and colleagues evaluated 10 autopsy adrenal glands and found that these organs were infiltrated by T-lymphocytes (CD3^+^ and CD8^+^ cells) [36].

Regarding other possible AI mechanisms in COVID-19, this virus expresses amino acids similar to human ACTH, and the antibody production by the immune system might impair endogenous ACTH function [32, 37]. Another possibility might be related to cytokines. It has been reported that tumor necrosis factor-*α* (TNF-*α*) reduces ACTH release and decreases its effect on adrenal tissue. Moreover, the pituitary gland is not protected by the blood-brain barrier, and extensive cytokine production (interleukin (IL) 1, IL 6, TNF-*α*, monocyte chemoattractant protein 1 (MCP1), and granulocyte-colony stimulating factor (G-CSF)) during COVID-19 infection might lead to hypothalamic-pituitary-adrenal axis dysfunction [38–41].

The anatomical location of the adrenal gland's vasculatures might be another culprit. These two tiny organs have a network of arterioles that arises from different main arteries, but blood drainage is limited due to a solitary suprarenal vein [42]. The high stress from COVID-19 infection might be a limiting factor for venous drainage due to ACTH-induced arteriolar dilation, leading to vascular stasis and subsequent adrenal damage [43].

Also, the severe status of the disease might be categorized as another explanation in AI incidence. Cholesterol, in the main form of high-density lipoprotein (HDL), is one of the main substrates for cortisol synthesis, and decreased HDL levels in severe disorders might lead to AI [44]. Furthermore, functional AI, named CIRCI, might also result from severe diseases. This functional AI is mainly due to inadequate cortisol secretion for the modulation of inflammatory responses. Multiple mechanisms, including reduced albumin or cortisol binding globulins (CBGs), decreased affinity, and reduced number of adrenal glands cortisol receptors or increased activity of 11-*β* hydroxysteroid dehydrogenase type 2 results in cortisol inactivation are proposed in this regard [45]. Mao et al. reported CIRCI diagnosis in 6 out of 9 critically infected patients with COVID-19 [27]. Long-term studies are required to evaluate whether AI is temporary and related to CIRCI or it would be permanent.

**Figure 2:**
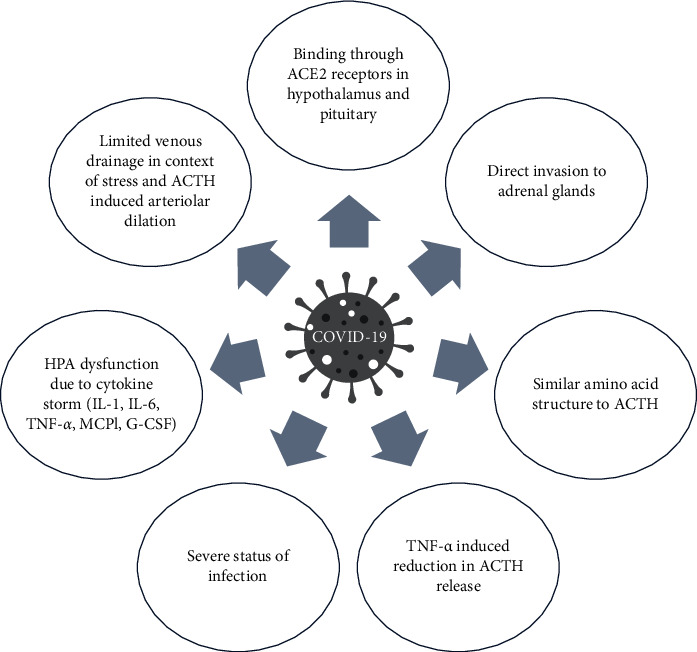
Summary of possible pathways of adrenal insufficiency in COVID-19 infection. ACE2: angiotensin-converting enzyme 2, ACTH: adrenocorticotropic hormone, TNF-*α*: tumor necrosis factor-*α*, IL: interleukin, MCP1: monocyte chemoattractant protein 1, and G-CSF: granulocyte-colony-stimulating factor.

Figure 2 provides a summary of all possible pathways of AI occurrence in the context of COVID-19 infection.

To the best of our knowledge, this review is the first in the literature to assess the status of AI occurrence in the COVID-19 era. By the way, several limitations are present. Due to daily reported information in this pandemic, we recruited all relevant records until May 23, 2021. Due to limited numbers of available records with high heterogeneity and a small sample size, we could not conduct a meta-analysis. Therefore, the generalization of our outcomes should be made with caution. Finally, we were unable to assess the frequency of AI based on disease severity.

In conclusion, we found that AI is a probable disease unrecognized in clinical settings in this COVID-19 pandemic. Therefore, assessing adrenal glands with laboratory or radiographic findings could be performed, especially among critically ill patients.

## Figures and Tables

**Table 1:** Summary of peer-reviewed cross-sectional studies reporting adrenal insufficiency in patients infected with COVID-19.

Authors	Date	Design	Sample size *N* (%)		Male (%)	Age (years)	Adrenal insufficiency assessment findings		Adrenal insufficiency type	Adrenal insufficiency frequency (%)
							Laboratory data	Radiographic findings		
Alzahrani et al. [25]	7 May –20 May 2020	Cross-sectional	Total	28 (100)	16 (57.1)	Median: 45.5 (range: 25–69)	Median cortisol: 196 (range: 31–587) nmol/l	NA	Central	Days 1–2: 18 out of 28 (64.3)
							Median ACTH: 18.5 (range: 4–38) ng/l			Days 3–5: 9 out of 20 (45)
							Cortisol< 100 nmol/l: 8 (28.6%)			
							Cortisol< 200 nmol/l: 14 (50%)			Days 8–11: 9 out of 15 (60)
							Cortisol< 300 nmol/l: 18 (64.3%)			
							ACTH<10 ng/l: 7 (26.9)			
							ACTH<20 ng/l: 17 (60.7%)			
							ACTH<30 ng/l: 23 (82.1%)			

Leyendecker et al. [26]	9 March –10 April 2020	Cross-sectional	Total	219 (100)	NA		Combination of hyperkalemia (>5 mmol/L), hyponatremia (<130 mmol/L), and hypoglycemia (<3.9 mmol/L)	Enlargement of adrenal glands with peripheral fat stranding in suprarenal region	NA	7 (3.1)
			Acute adrenal infarction	51 (23)	36 (70.5)	67 ± 11 (range: 42–88)				4 (7.8)
			No acute adrenal infarction	168 (77)	123 (73.2)	67 ± 15 (range: 22–96)				3 (1.7)

Mao et al. [27]	1 March –1 May 2020	Cross-sectional	Total	21 (100)	5 (23.8)	NA	Cortisol<10 *µ*g/dl	NA	NA	NA
			COVID-19 group	9 (42.9)	1 (11.1)	71.7 ± 8.1				6 (66.6)^*∗*^
			Non COVID-19	12 (57.1)	4 (33.3)	68.9 ± 20.2				NA

ACTH: adrenocorticotropic hormone; NA: not available. ^*∗*^Adrenal insufficiency was suggested to be in context of critical illness-related corticosteroid insufficiency.

**Table 2:** Summary of peer-reviewed case report studies reporting adrenal insufficiency in patients infected with COVID-19.

Authors	Sample size (*N*)	Sex	Age (years)	Presenting symptoms	Adrenal insufficiency assessment findings		Adrenal insufficiency type	Patient status	Follow-up assessment
					Laboratory data	Radiographic findings			
Alvarez-Troncoso et al. [24]	1	Male	70	Fever, chills, asthenia, constipation, malaise, weakness, anorexia, nausea, vomiting	NA	Increase in size and blurring of both adrenals	NA	Discharged	Cortisol Basal: 2.1 *µ*g/dl 30 minutes: 2.89 *µ*g/dl 60 minutes: 3.11 *µ*g/dl

Elkhouly et al. [28]	1	Male	50	Fever, malaise, shortness of breath, cough	NA	Bilateral hyperdense ovoid suprarenal lesions with loss of normal adrenal gland contour	NA	Death	—

Frankel et al. [29]	1	Female	66	Fever, dyspnea, nausea, vomiting, abdominal pain	Baseline cortisol< 1 *µ*g/dl ACTH: 207 pmol/l	Enlarged and thick adrenal glands plus haziness of surrounding peri-adrenal fat	Primary	Discharged	Stable

Hashim et al. [17]	1	Male	51	Vomiting	Cortisol Baseline: 56 nmol/l 30 minutes: 197 nmol/l 60 minutes: 297 nmol/l	NA	NA	Discharged	Lost
Heidarpour et al. [18]	1	Male	69	Fever, dyspnea, cough	Total cortisol< 12 *µ*g/dl	NA	Primary	Discharged	NA

Kumar et al. [30]	1	Female	70	Fever, left-sided chest pain, cough, dyspnea, fatigue, abdominal pain, vomiting, diarrhea	Random cortisol> 300 nmol/l	Enlarged diffusely hypoattenuated adrenal glands with poor enhancement and ill-defined adrenal contours	NA	Discharged	NA

Sheikh et al. [31]	1	Female	44	Fever, chills, malaise, shortness of breath, myalgia, loss of taste	Random cortisol: 1.1 *µ*g/dl, ACTH: 56 pg/ml	NA	Central	Discharged	Lost

ACTH: adrenocorticotropic hormone; NA: not available.

## Data Availability

The datasets generated during and/or analyzed during the current study are not publicly available due to confidential issues but are available from the corresponding author on reasonable request.

## Abbreviations:

SARS: Severe acute respiratory syndrome
MERS: Middle East respiratory syndrome
COVID-19: Coronavirus disease 2019
WHO: World Health Organization
ACE2: Angiotensin-converting enzyme 2
AI: Adrenal insufficiency
PRISMA: Preferred Reporting Items for Systematic Reviews and Meta-Analysis
PROSPERO: International Prospective Register of Systematic Reviews
AMSTAR: Assessment of multiple systematic reviews
AXIS: Critical appraisal tool for cross-sectional studies
JBI: Joanna Briggs Institute (JBI)
NIH: National Institutes of Health
CT: Computed tomography
ACTH: Adrenocorticotropic hormone
CIRCI: Critical illness-related corticosteroid insufficiency
TNF-*α*: Tumor necrosis factor-*α*
IL: Interleukin
MCP1: Monocyte chemoattractant protein 1
G-CSF: Granulocyte-colony-stimulating factor
HDL: High-density lipoprotein
CBG: Cortisol binding globulins.

## Supplementary Materials

Supplementary Materials Table S1: risk of bias assessment of cross-sectional studies. Table S2: risk of bias assessment of case report studies.
