# Supplementary material for: Adrenal Insufficiency in Coronavirus Disease 2019 (COVID-19)-Infected Patients without Preexisting Adrenal Diseases: A Systematic Literature Review
Source: Int J Endocrinol. 2021 Sep 14;2021:2271514. doi: 10.1155/2021/2271514 (PMC8443384; doi:10.1155/2021/2271514)
Supplement: Supplementary Materials — Table S1: risk of bias assessment of cross-sectional studies. Table S2: risk of bias assessment of case report studies. [file 2271514.f1.docx]

**Supplementary materials**

**Table S1. Risk of bias assessment of cross-sectional studies**

| Components | Alzahrani et al. | Leyendecker et al. | Mao et al. |
| --- | --- | --- | --- |
| 1. Were the aims/objectives of the study clear? | Yes | Yes | Yes |
| 2. Was the study design appropriate for the stated aim(s)? | Yes | Yes | Yes |
| 3. Was the sample size justified? | No | No | No |
| 4. Was the target/reference population clearly defined? (Is it clear who the research was about?) | Yes | Yes | Yes |
| 5. Was the sample frame taken from an appropriate population base so that it closely represented the target/reference population under investigation? | Yes | Yes | Yes |
| 6. Was the selection process likely to select subjects/participants representing the target/reference population under investigation? | Yes | Yes | Yes |
| 7. Were measures undertaken to address and categorize non-responders? | Not applicable | Not applicable | Not applicable |
| 8. Were the risk factor, and outcome variables measured appropriate to the aims of the study? | Yes | Yes | Yes |
| 9. Were the risk factor and outcome variables measured correctly using instruments/ measurements that had been trialed, piloted, or published previously? | Yes | Yes | Yes |
| 10. Is it clear what was used to determining statistical significance and/or precision estimates? (e.g., p values, CIs) | Yes | Yes | Yes |
| 11. Were the methods (including statistical methods) sufficiently described to enable them to be repeated? | Yes | Yes | Yes |
| 12. Were the basic data adequately described? | Yes | Yes | Yes |
| 13. Does the response rate raise concerns about non-response bias? | No | No | No |
| 14. If appropriate, was information about non-responders described? | Not applicable | Not applicable | Not applicable |
| 15. Were the results internally consistent? | Yes | Yes | Yes |
| 16. Were the results for the analyses described in the methods presented? | Yes | Yes | Yes |
| 17. Were the authors’ discussions and conclusions justified by the results? | Yes | Yes | Yes |
| 18. Were the limitations of the study discussed? | Yes | Yes | Yes |
| 19. Were there any funding sources or conflicts of interest that may affect the authors’  interpretation of the results? | No | No | No |
| 20. Was ethical approval or consent of participants attained? | Yes | Yes | Yes |

**Table S2. Risk of bias assessment of case report studies**

| Components | Alvarez-Troncoso et al. | Elkhouly et al. | Frankel et al. | Hashim et al. | Heidarpour et al. | Kumar et al. | Sheikh et al. |
| --- | --- | --- | --- | --- | --- | --- | --- |
| 1. Were the patient’s demographic characteristics clearly described? | Yes | Yes | Yes | Yes | Yes | Yes | Yes |
| 2. Was the patient’s history clearly described and presented as a timeline? | Yes | Yes | Yes | Yes | Yes | Yes | Yes |
| 3. Was the current clinical condition of the patient on presentation clearly described? | Yes | Yes | Yes | Yes | Yes | Yes | Yes |
| 4. Were diagnostic tests or assessment methods and the results clearly described? | Yes | Yes | Yes | Yes | Yes | Yes | Yes |
| 5. Was the intervention(s) or treatment procedure(s) clearly described? | Yes | Yes | Yes | Yes | Yes | Yes | Yes |
| 6. Was the post-intervention clinical condition clearly described? | Not applicable | Not applicable | Not applicable | Not applicable | Not applicable | Not applicable | Not applicable |
| 7. Were adverse events (harms) or unanticipated events identified and described? | Not applicable | Not applicable | Not applicable | Not applicable | Not applicable | Not applicable | Not applicable |
| 8. Does the case report provide takeaway lessons? | Yes | Yes | Yes | Yes | Yes | Yes | Yes |
| Overall appraisal: | Include | Include | Include | Include | Include | Include | Include |
